# Induction of endogenous retroelements as a potential mechanism for mouse-specific drug-induced carcinogenicity

**DOI:** 10.1371/journal.pone.0176768

**Published:** 2017-05-04

**Authors:** Timothy M. Coskran, Zhijie Jiang, James E. Klaunig, Dixie L. Mager, Leslie Obert, Andrew Robertson, Nicholas Tsinoremas, Zemin Wang, Mark Gosink

**Affiliations:** 1 Drug Safety Research & Development, Pfizer Inc., Groton, Connecticut, United States of America; 2 Department of Computer Science, University of Miami, Miami, Florida, United States of America; 3 Environmental Health, Indiana University, Bloomington, Indiana, United States of America; 4 Terry Fox Laboratory, BC Cancer Agency, Vancouver, British Columbia, Canada; 5 GlaxoSmithKline plc, King of Prussia, Pennsylvania, United States of America; Universite Paris-Sud, FRANCE

## Abstract

A number of chemical compounds have been shown to induce liver tumors in mice but not in other species. While several mechanisms for this species-specific tumorigenicity have been proposed, no definitive mechanism has been established. We examined the effects of the nongenotoxic rodent hepatic carcinogen, WY-14,643, in male mice from a high liver tumor susceptible strain (C3H/HeJ), and from a low tumor susceptible strain (C57BL/6). WY-14,643, a PPARα activator induced widespread increases in the expression of some endogenous retroelements, namely members of LTR and LINE elements in both strains. The expression of a number of known retroviral defense genes was also elevated. We also demonstrated that basal immune-mediated viral defense was elevated in C57BL/6 mice (the resistant strain) and that WY-14,643 further activated those immuno-defense processes. We propose that the previously reported >100X activity of retroelements in mice drives mouse-specific tumorigenicity. We also propose that C57BL/6’s competent immune to retroviral activation allows it to remove cells before the activation of these elements can result in significant chromosomal insertions and mutation. Finally, we showed that WY-14,643 treatment induced gene signatures of DNA recombination in the sensitive C3H/HeJ strain.

## Introduction

Pharmaceutical and non-pharmaceutical chemicals have been shown to produce liver tumors in mice, which function through non DNA reactive (nongenotoxic) mechanisms [1]. These nongenotoxic mechanisms are often species and tissue specific and most times do not translate to a human liability [2–4]. However, a positive signal for carcinogenicity in a 2-year carcinogenicity study in rodents often results in significant development program delays and costs to the program. In numerous cases, the responsible regulatory agency has required additional experiments be performed to better elucidate the underlying mechanism of the tumorigenesis and the relevance of the tumor finding in mice to potential human risk.

Mice have a relatively high spontaneous rate of liver tumors which has been linked to an increased sensitivity to liver carcinogens. The relevance of mouse liver tumors to human risk has been studied extensively [3, 5–7]. A number of mechanisms have been described for liver specific rodent nongenotoxic carcinogenesis [6, 7]. In recent years the role of histone and DNA methylation as related to epigenetic changes following drug treatment has been studied [8, 9]. The compound WY-14,643 is a well-studied rodent liver carcinogen that functions through nongenotoxic mechanisms. Several groups have also shown that WY-14,643 induces a number of chromatin effects including DNA and histone demethylation. In one report, Pogribny et al, demonstrated that long-term treatment of mice with WY-14,643 results in significant decreases in histone H3 lysine 9 trimethylation and the demethylation of a number of retroelements [10].

Karimi et al showed that complete loss of H3K9me3 resulted in the dramatic transcriptional de-repression of numerous retroelement families [11]. Retro-transposition is known to play a significant role in mouse mutagenesis, where it accounts for ~10% of all spontaneous mutations [12]. This rate is at least 100 times that which occurs in humans where endogenous retroviruses are likely incompetent for retrotransposition [13]. As such, drug-induced retro-transposition or retroelement-mediated gene expression changes may represent as of yet unexplored avenues for the induction of many mouse-specific tumors.

Compounds that alter the activity of epigenetic modifying enzymes might cause reactivation of endogenous retroelements and latent viruses could increase risk of cancer. There are numerous reports that indicate the latency of viruses, such as Epstein—Barr Virus, Herpes Simplex Virus, Karposi’s Sarcoma Virus and Human Immune-deficiency Virus (HIV), is regulated by histone modification and DNA methylation status [14–17]. Some groups have also reported that retro-transpositional events have been seen in association with other human diseases, including some cancers [18].

To examine the role that retroelement activation plays in mouse-specific carcinogenesis, in the current study we examined the expression profiles of two strains of mice, C3H/HeJ and C57BL/6. The C3H/HeJ mouse has a high spontaneous liver tumor incidence and is highly susceptible to liver tumor induction following exposure to rodent hepatic carcinogens. In contrast, the C57BL/6 mouse has a much lower spontaneous liver tumor incidence and is significantly less susceptible to rodent hepatic carcinogens. Using RNA-SEQ analysis to examine all expression originating from the 5.1 million genomic repetitive elements (retroelements), we found that WY-14,643 induces the expression of retroelements known to have mutagenic potential. Subsequent gene expression and pathway analyses are consistent with retroelement activation. We also used pathway analyses as well as histologic staining to examine the difference between the tumor susceptible and tumor-resistant strains. The results support a strong antiviral immune response in the resistant C57BL/6 strain. We propose that WY-14,643 induces retroviral mediated mutagenesis and that strain to strain susceptibility is due to variation in immune response to viral activation. The proposed mechanism is consistent with the work of others who have shown that that WY-14,643 induces DNA demethylation [10] and that DNA demethylation can lead to retroelement activation and mutagenesis in mice [19, 20].

Given the broad range of compounds known to effect DNA methylation states and the inherent susceptibility of mice to retroelement driven carcinogenicity, the proposed mechanism could explain a number of instances where there is an apparent mouse-specific carcinogenicity observed with some compounds.

## Materials and methods

### Animal studies

Male C3H/HeJ and C57BL/6 mice (7 weeks old) were purchased from Harlan Sprague-Dawley Co. (Indianapolis, IN). Mice were housed in the AAALAC certified animal facility at Indiana University, Bloomington, Indiana. All animals were maintained in accordance to the Guide for the Care and Use of Laboratory Animals from the Institute of Laboratory Animal Research, and approved by the Institutional Animal Care and Use Committee at Indiana University. The Indiana University Bloomington campus’ animal facility approved by AAALAC provides housing for different species of experimental animals. Mice were housed (five per cage) in individually ventilated cages under conditions of controlled temperature (22 ± 1°C), humidity (40–70%), and light cycle (12 hr/12 hr), and were given food and municipal water *ad libitum*. All animals were acclimated for 7 days prior to the initiation of the study. Individual mice were ear-tagged for identification throughout the study and randomly assigned to control or exposure groups (5/group). WY-14,643 was administered in powder diet that was formulated by mixing WY-14,643 stock solution (Sigma, St Louis, MO) with the premix AIN76a diet (Dyets Inc., Bethlehem, PA). Mice (n = 5/dose group) were treated with WY-14,643 at 1000 ppm in diet or control diet (corn oil) for 4 weeks and were sacrificed by CO2 asphyxiation. The body weight and liver weight were recorded. Blood was collected through cardiac puncture. A necropsy was performed with particular attention to liver lesions. A portion of liver from each animal was fixed in 10% neutral buffered formalin for histopathology examination by hematoxylin and eosin (H&E) staining. The rest of the liver was snap frozen and stored in -80°C freezer for RNA isolation and transcriptome sequencing analysis.

### RNA-SEQ

Liver samples were homogenized in Qiagen’s Qiazol tri-reagent using molecular homogenization tubes (M-tubes) in a Miltenyi Biotec GentleMACS system. Total RNA was extracted using Qiagen’s miRNeasy mini kit per manufacturer's instructions. Samples were treated with Ambion DNA-free DNAse and integrity was assessed using an Agilent Bioanalyzer 2100 with RNA nano 6000 chips. Ribo-Depletion of total RNA was performed using the Epicentre Ribo-Zero^™^ Gold Kit (part# RZHM11106) followed by library construction of samples using the Epicentre ScriptSeq^™^ v2 RNA-Seq Library Preparation Kit (part# SSV21124) according to manufacturer’s protocols from 800ng total RNA via Agilant Bioanalyzer and 12 cycles of PCR. Sequencing was performed on an Illumina HiSeq2000 using the reagents provided in the TruSeq PE Cluster Kit v3-cBot-HS (Catalog #: PE-401-3001) and the TruSeq SBS Kit v3-HS (Catalog #: FC-401-3001) kit. Paired-end reads of ~100 bp length were performed to a depth of ~35 million reads per sample.

### Transcriptional profiling

In three samples, insufficient reads were obtained from the first run so samples were re-sequenced. Another sample was accidently sequenced twice. In both cases, second runs were concatenated with the first runs before analyses. Sample reads were aligned to the mm10 version of the mouse genome using STAR version STAR_2.4.0h [21]. Default parameters for STAR were used except for the following: “—alignSJDBoverhangMin 1—outSAMstrandField intronMotif—outFilterMismatchNoverLmax 0.1—alignIntronMax 1000000—outFilterMultimapNmax 1000”. SAMtools version 0.1.18 were used for post-alignment processing [22]. For analyses of the retroelements, a file of mouse retroelements was generated by extracting mapping locations and annotations from the rmsk.txt file from the UCSC mm10 goldenPath (ftp://hgdownload.cse.ucsc.edu/goldenPath/mm10/database/) and converted into gtf format. Fragments Per Kilobase of transcript per Million (FPKM) values were calculated using cufflinks version v2.0.2 [23]. For analyses of mouse genes, transcript splice forms were identified using Spanki version 0.4.0 [24]. Genes were annotated and gene counts generated using the intersectBed application from BedTools version 2.18.2 [25]. Reads Per Kilobase of transcript per Million (RPKM) values were calculated with a script from transcript sizes.

### Analysis of gene expression data

Of the 5,138,235 annotated retroelements, the 1,060,164 which contained at least one non-zero FPKM sample value were retained for further analysis. Mann-Whitney tests were used to calculate p-values for comparisons of treated samples to the controls of the same strain. Retroelements which had p-values less than 0.05 either in the C3H/HeJ comparisons or in the C57BL/6 comparisons were used for analysis. Average values for each strain-treatment group were calculated.

For gene expression analyses, genes which had Mann-Whitney test values less than 0.05 and absolute fold-change values greater than 1.5X were used for subsequent analyses in QIAGEN’s Ingenuity^®^ Pathway Analysis (IPA^®^, QIAGEN Redwood City, www.qiagen.com/ingenuity). Briefly, Ingenuity maintains a large repository of annotated biochemical and regulatory pathways. They also curate collections of gene-to-gene regulatory effects as well as annotated sets of genes with common roles in biologic functions and toxicities. Typically Ingenuity analyses will be scored two ways. The first is a p-value which is the probability that a given list of genes (i.e. a list of differentially expressed genes, DEGs) would coincide with Ingenuity’s gene sets. The second is the activation z-score that is a correlation measure of how consistent the direction of expression changes in submitted DEG list matches the change direction from the literature for most targets in the biologic or regulatory group.

### Histology

Tissue Processing and Staining: Formalin fixed liver samples were prepared for histology in paraffin-embedded blocks by AML Laboratories Inc. (Baltimore, MD) according to their standard protocol. Blocks were cut at 5 microns, dried overnight, and stained with hematoxylin and eosin (H&E) using the Ventana Symphony^®^(Ventana Medical Systems, Tucson, AZ) slide stainer using the system’s reagents according to manufacturer’s directions.

*Histopathology Grading*: Microscopic examination was performed. Three lesions were identified in the livers of mice (hepatocyte hypertrophy, hepatocyte mitotic figures and sinusoidal cellular infiltrate). Each of these changes were graded as minimal (1), mild (2), moderate (3), or marked (4). Grade 1—Minimal: a histopathologic change ranging from inconspicuous to barely noticeable but so minor, small, or infrequent as to warrant no more than the least assignable grade. Grade 2—Mild: a histopathologic change that is a readily noticeable but not a prominent feature of the tissue and/or may be considered to be of no functional consequence. Grade 3—Moderate: a histopathologic change that is a prominent but not a dominant feature of the tissue and/or may be considered to have limited impact on organ function. Grade 4—Marked: a histopathologic change that is a dominant feature and may be an overwhelming component of the tissue and/or may cause significant tissue or organ dysfunction.

## Results

### Retroelement expression

Individual retroelement loci which were differentially expressed between treatment and controls were identified by selecting for those with p-values ≤ 0.05 in one or both of the treatment comparisons. In both strains, WY-14,643 treatment resulted in significant overall increased in the expression of LTR and LINE retroelements (Table 1). This is consistent with the known demethylating effects of WY-14,643 on the chromosome and the known effects of hypomethylation on gene expression [10]. The exact retroelements with altered expression varied widely between strains. For example, C3H/HeJ and C57BL/6 showed altered expression in 957 and 1414 LTR and 1547 and 2301 LINE retroelements, respectively. However, C3H/HeJ and C57BL/6 had only 85 & 168 shared retroelements with altered expression. Closer examination of the average expression of the most retrotranspositionally active LTR families [12] shows a non-significant trend towards increased expression with WY-14,643 treatment (Table 1), suggesting that relatively few members of these families are transcriptionally upregulated.

**Table 1:** Differentially expressed retroelements between treatment and controls.

Elements		C3H Untreated	C3H Treated	C57 Untreated	C57 Treated
LINEs	Average FPKM	0.314	0.653	0.329	0.619
	p-value	0.036		0.020	
LTRs	Average FPKM	0.386	0.767	0.459	0.911
	p-value	0.025		0.020	
ETn	Average FPKM	0.2157	0.3806	0.3400	0.6144
	p-value	0.1211		0.0853	
IAP	Average FPKM	0.0599	0.3406	0.0983	0.1231
	p-value	0.2464		0.3883	
MERV-L	Average FPKM	0.1106	0.2262	0.1544	0.1531
	p-value	0.4145		0.9879	
VL-30	Average FPKM	2.5049	8.0256	3.1259	13.0507
	p-value	0.3099		0.1158	

**Fig 1:**
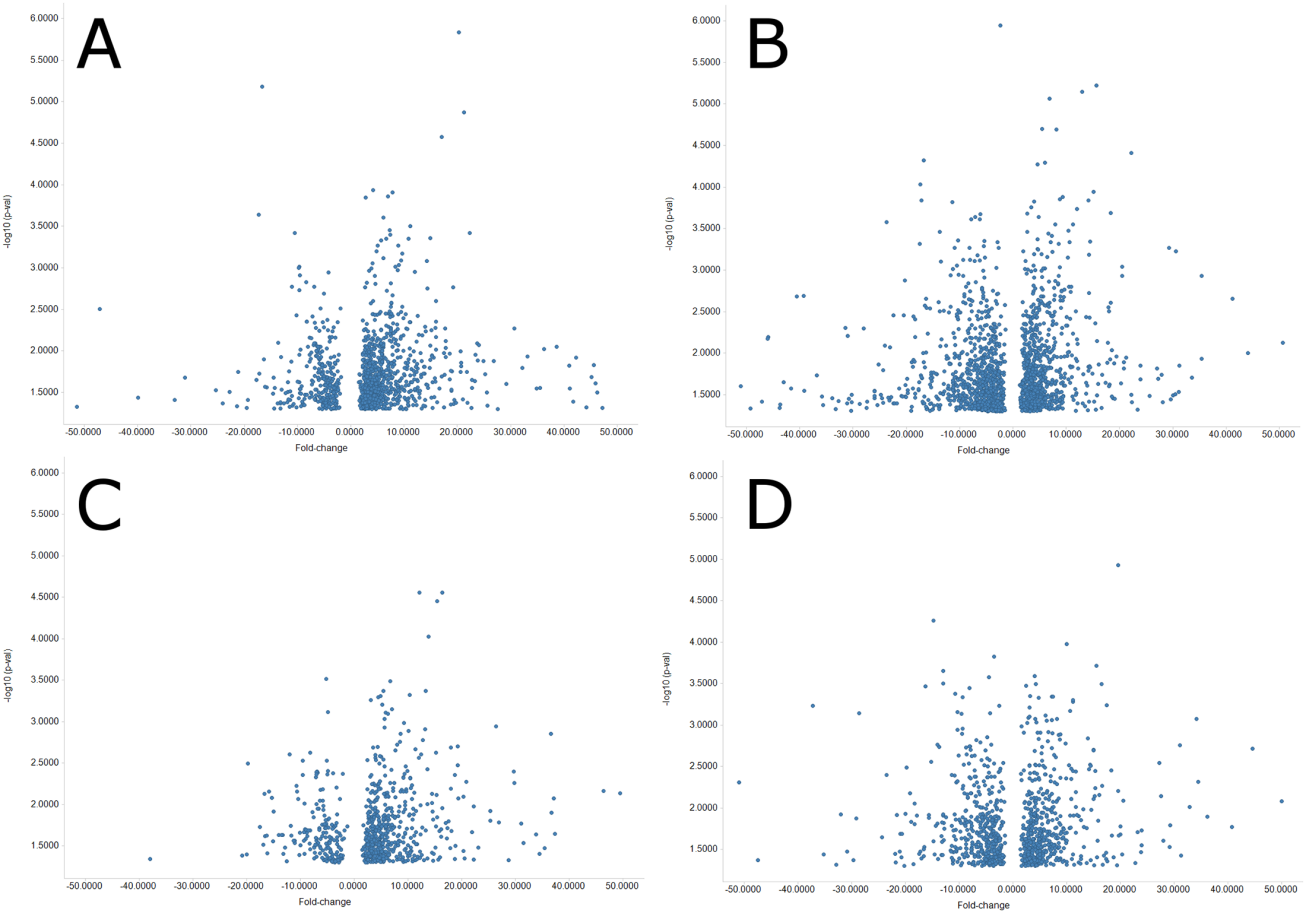
Volcano plot of LINE and LTR elements. Panels A and B show fold-change versus -log10 of p-value of LINE elements which show significant change in the respective strains. Panels C and D show fold-change versus -log10 of p-value for the LTR elements. Panels A and C show elements significantly changed in the C3H/HeJ strain while panels B and C show elements from the C57BL/6 strain. Panels were clipped at plus/minus 50X fold-change for clarity resulting in 15, 14, 4, & 5 outliers not being shown on panels A, B, C, & D respectively. Unclipped figures are available in the supplementary files (S1 Excel File).

We also examined the numbers of retroelements with significant changes in expression with treatment for each strain (Fig 1). For the C3H/HeJ strain, proportionality testing indicates that there is a significant bias towards LTR and LINE elements with increased expression (Table 2). In the C57BL/6 strain, there were significantly more LINE elements with decreased expression (Table 2). The number of LTR increasing was roughly equivalent to the number decreasing. A breakdown of the most retrotranspositionally active LTR families reveals a significant trend towards increased expression of these elements in the C3H/HeJ strain while a mixed trend was seen in the C57BL/6 strain. It should be noted that we may be underestimating the number of the youngest retroelements. The youngest elements tend to be the most retranspositionally active and have also not had the evolutionary time for their sequence to diverge. While we attempted to allow sequencing reads to map to up to 1000 locations, highly active elements could exceed this number. Additionally, many LTR elements, particularly IAPs, in C3H/HeJ are not even present in C57BL/6 so reads to those elements will also be unable to be mapped to the reference C57BL/6 genome [26].

**Table 2:** Expression pattern of retroelements in two strains.

Elements		C3H Decreasing	C3H Increasing	C57 Decreasing	C57 Increasing
LINEs	Count	449	1098	1356	945
	p-value	<2.2e-16		<2.2e-16	
LTRs	Count	271	686	722	692
	p-value	<2.2e-16		0.44	
ETn	Count	3	8	7	10
	p-value	0.23		0.63	
IAP	Count	15	41	72	33
	p-value	8.4e-4		2.1e-4	
MERV-L	Count	7	12	31	13
	p-value	0.36		0.01	
VL-30	Count	2	26	3	51
	p-value	1.4e-5		1.6e-10	

**Fig 2:**
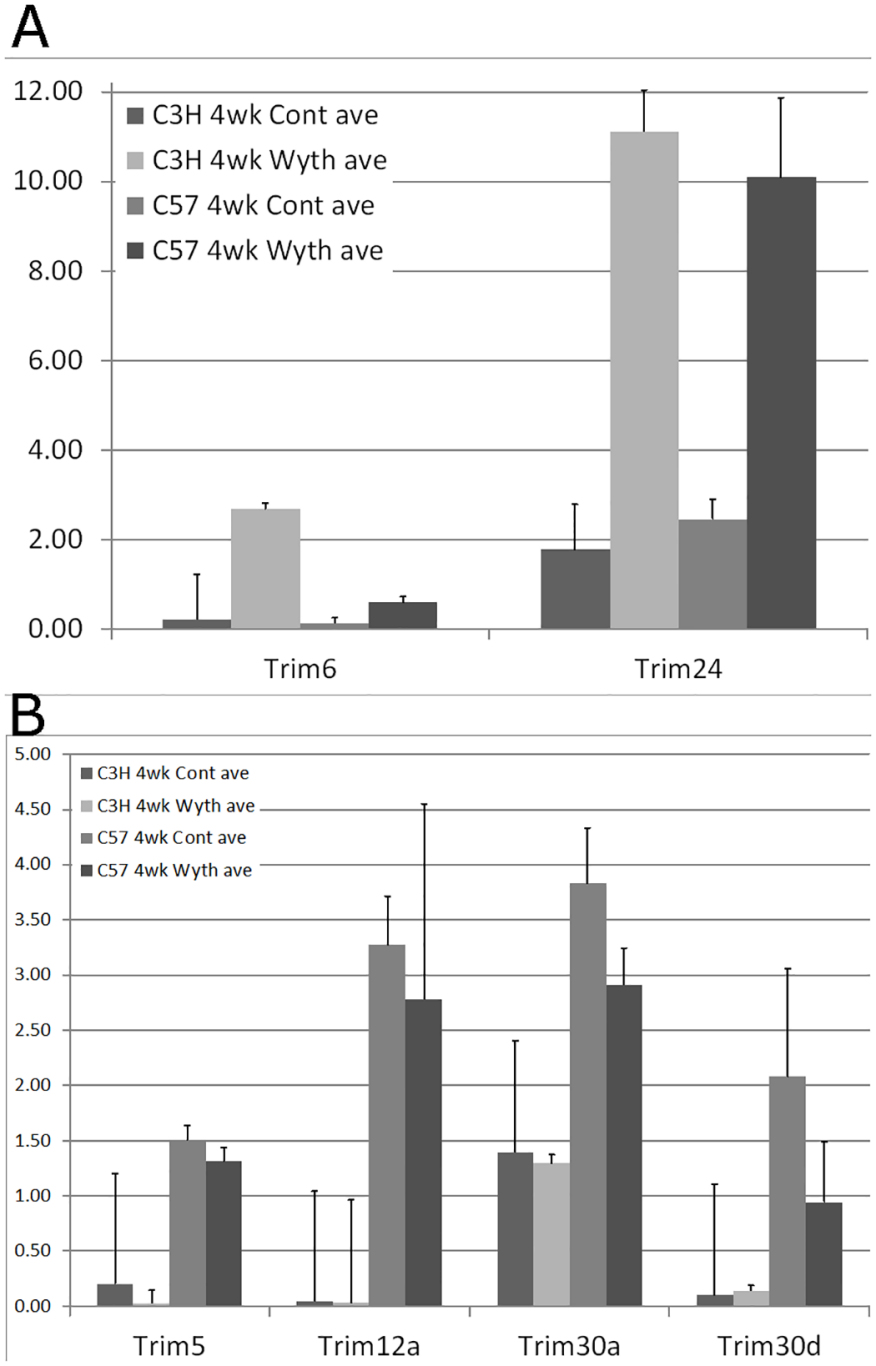
Expression of anti-retroviral TRIM genes. Panel A) Expression of Trim6 and Trim24 genes which show significant expression increases with WY-14,643 treatment. Panel B) Expression of Trim5, Trim12a, Trim30a and Trim30d genes in the C3H/HeJ and C57BL/6 treatment groups. Genes exhibit significant expression increases in C57BL/6 samples as compared to the C3H/HeJ.

Cells have a variety of intrinsic mechanisms to protect themselves from retroviral activity [27, 28]. One important family of genes in this defense is the tripartite motif (TRIM) proteins, which are up-regulated in response to viral induction of interferon [29, 30]. Two of these genes, Trim6 (C3H:5.6e-3, C57:1.6e-3) and Trim24 (C3H:3.1e-3, C57:2.3e-3) show significant expression increases in response to WY-14,643 treatment further supporting the treatment induced activation of retroviral activity (Fig 2A).

### Basal strain differences in viral immune functionality

**Fig 3:**
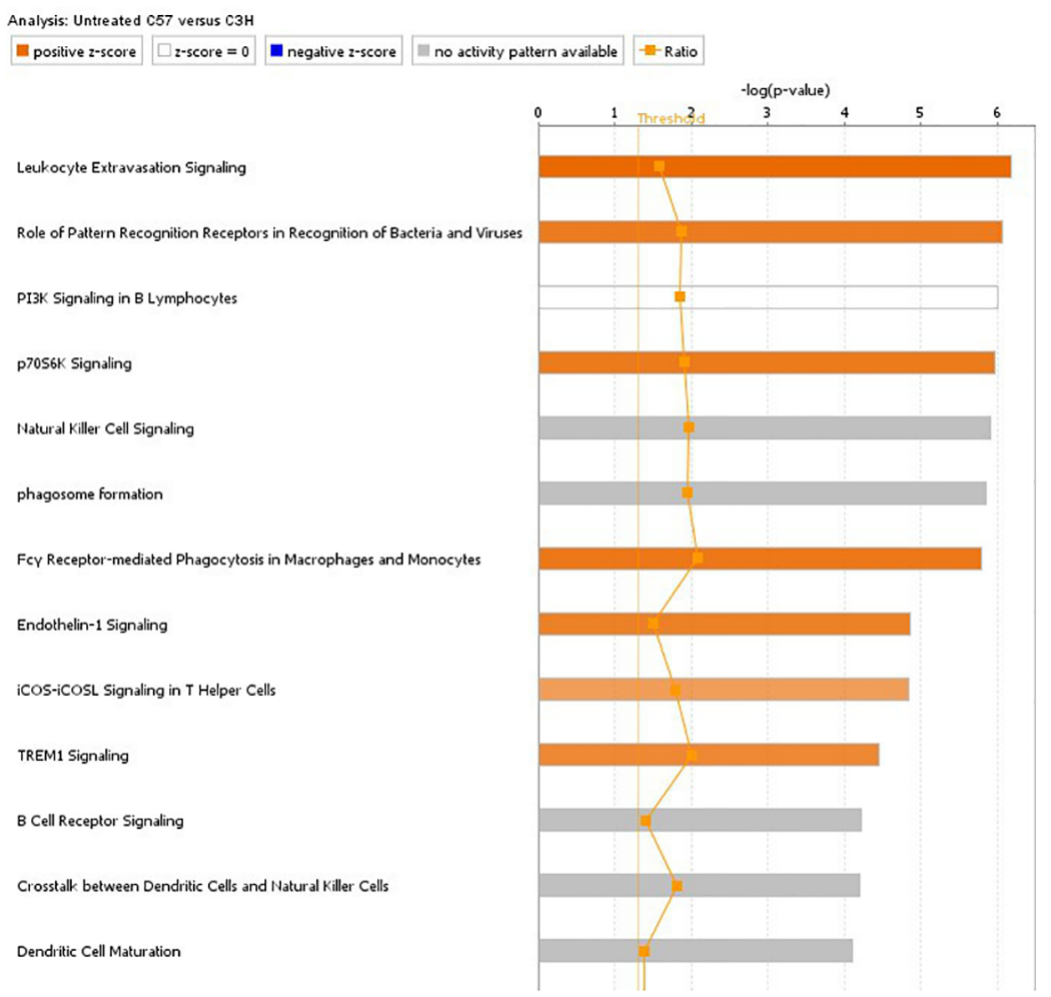
Ingenuity pathway analysis of genes differentially expressed between untreated C57BL/6 and C3H/HeJ samples. Bar length is proportional to -log10 of p-value. Orange color indicates activation of the pathway, blue indicates suppression of the pathway and grey indicates a mixed response.

Pathway analyses of the differentially expressed genes between the untreated strain samples revealed a fundamental and significant difference in many immune related pathways and functions (Fig 3 and S2 Excel File). In addition, Upstream Regulator Analysis reveals the activity of many immune-regulatory factors show significantly increased activity in C57BL/6 mice. Amongst the top regulators showing increased activity are interferon gamma, the interferon alpha/beta group, IL10 and tumor necrosis factor (S2 Excel File). All of these genes are deeply tied to the body’s response to retroviral infection and are also known to be stimulated by TLR4 activation [31–35]. In addition, we found the expression of several host restriction factors, including Trim5, Trim12a, Trim30a, and Trim30d, to be significantly elevated (Trim5:2.4e-5, Trim12a:1.2e-5, Trim30a:1.9e-5, Trim30d:6.9e-4) in the C57BL/6 strain samples (Fig 2B). These results support the hypothesis that C57BL/6 mice are more immune-competent to respond to activation of endogenous retroviruses. An active response to basal retrovirus is supported by the increased “poly rI:rC-RNA” activity seen in the analysis (S2 Excel File). Poly I:C is often used as a model of double stranded RNA and viral infection to trigger an immune response via pattern-recognition receptors [36, 37].

### WY-14,643-treatment of C57BL/6

**Fig 4:**
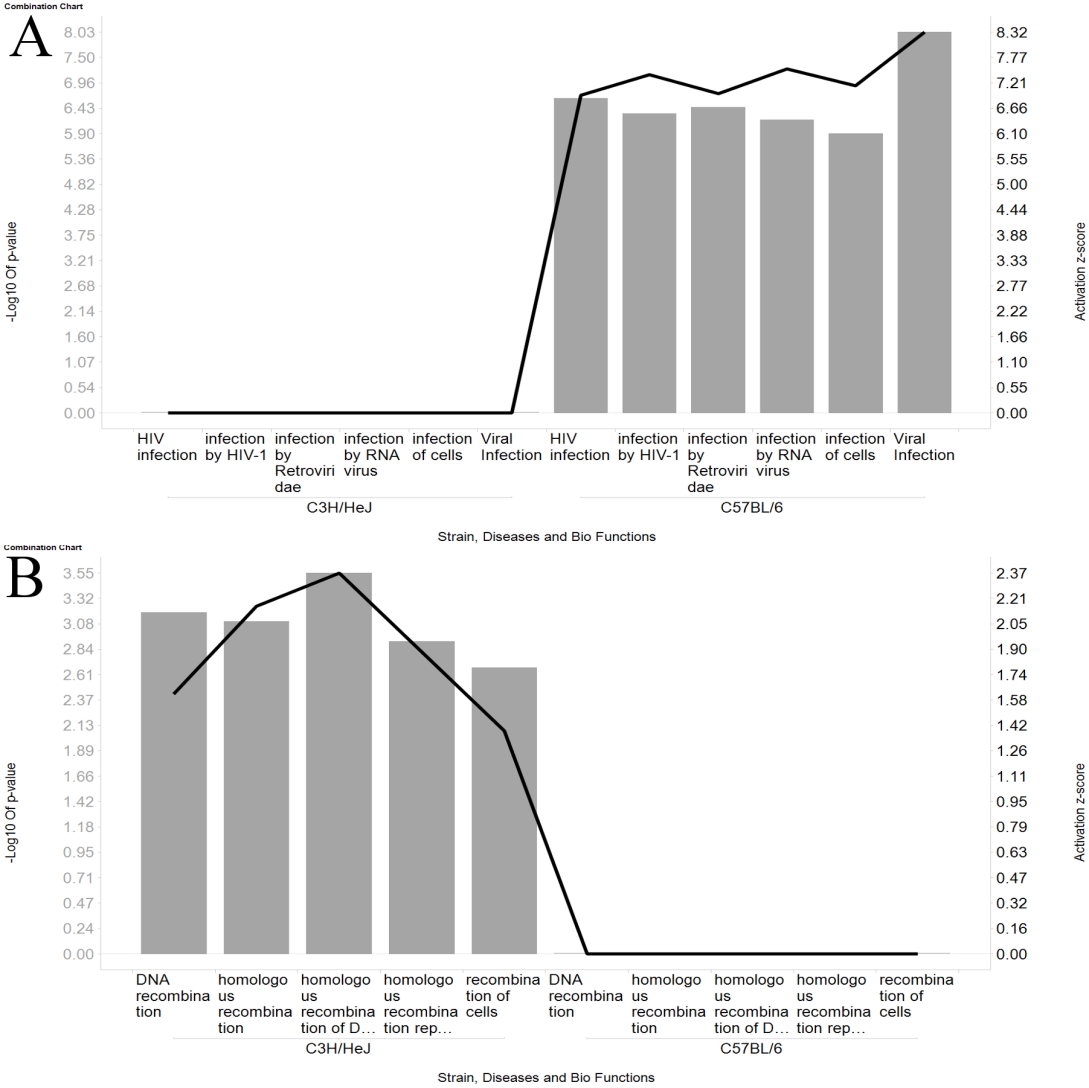
Ingenuity biofunction analysis results for selected categories. The gray bars are proportional to the -log10 of the function p-values and the black line indicates the Activation z-scores. Results compare the functions predicted for WY-14,643 treatment of the C3H/HeJ animals with the treatment of the C57BL/6 animals. Panel A compares several of the most significant viral biofunctions identified by Ingenuity’s Bio & Tox Function analysis. Panel B compares the significant biofunctions related to recombination in the chromosomes.

Diseases and Bio Functions analysis revealed that the most of the observed gene changes in WY-14,643 treated C57BL/6 samples were related to the PPARα agonist activity of this compound. For example, the predominant functions were around lipid and cholesterol metabolism (S3 Excel File). However, functions related to “Viral Infection” are also strongly activated in response to treatment. Within “Viral Infection”, the sub-function “infection by Retroviridae” has a very significant overlap p-value of 2.3e-7 and also a high activation z-score indicating that within the differentially expressed genes in the C57BL/6 strain are a significant number of genes related to this sub-function but also their direction of change is highly consistent with increasing activity (Fig 4A).

The “Mitochondrial Dysfunction” canonical pathway was significantly altered (p-value: 2.76e-12) in the C57BL/6 WY-14,643 treated samples compared to the C3H/HeJ WY-14,643 treated (p-value: 1.81e-2) (S3 Excel File). This result is consistent with the central role that mitochondria have in innate antiviral immune response [38, 39].

Support for C57BL/6’s increased antiviral response is provided by Upstream Analysis which indicated WY-14,643 treatment further increased the activity of IFNG & TNF (S3 Excel File). In addition, Interferon and TGFβ activity are shown to be increased by “Upstream Regulator” analysis (S3 Excel File).

**Fig 5:**
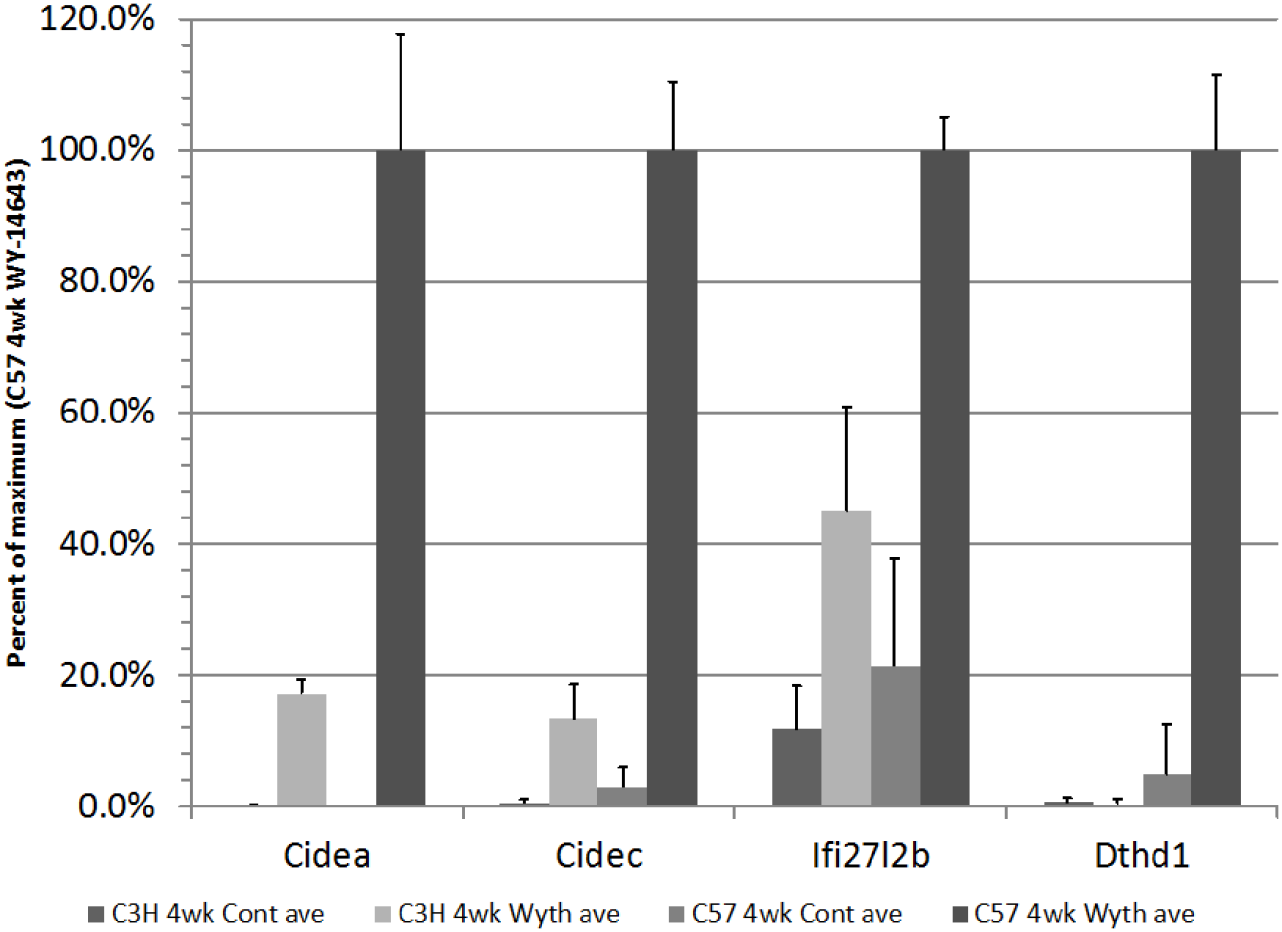
Expression of genes related to cell-death induction. Expression values are normalized to their expression in the WY-14,643-treated C57BL/6 strain. Error bars indicate standard deviation.

We also observed a significant elevation (4.6 fold; p-val = 1.29e-3) in the expression of Ifi27l2b (aka Isg12b2). Ifi27l2b is an interferon-inducible gene whose role has been shown to mediate virus-induced cell death [40]. In accordance with the role of Ifi27l2b, we saw significant elevations of the cell death-inducing DFFA-like effectors A & C (Cidea & Cidec) as well as death domain containing 1 (Dthd1) (Fig 5).

### WY-14,643-treatment of C3H/HeJ

As with C57BL/6, analysis of WY-14,643-treatment induced gene changes in C3H/HeJ with “Diseases and Bio Functions” indicates that the predominant activity increases are related to the therapeutic role of PPARα agonists with lipid and cholesterol functions (S4 Excel File). None of the “Viral Infection” functions observed with C57BL/6 were identified as significant within the differentially expressed genes from the WY-14,643-treated C3H/HeJ strain samples. However, consistent with an unchecked retroelement population, “Diseases and Bio Functions” analysis reveals significant increases in genetic recombination (Fig 4B and S4 Excel File).

### WY-14,643-treated C3H/HeJ versus C57BL/6 histology

**Fig 6:**
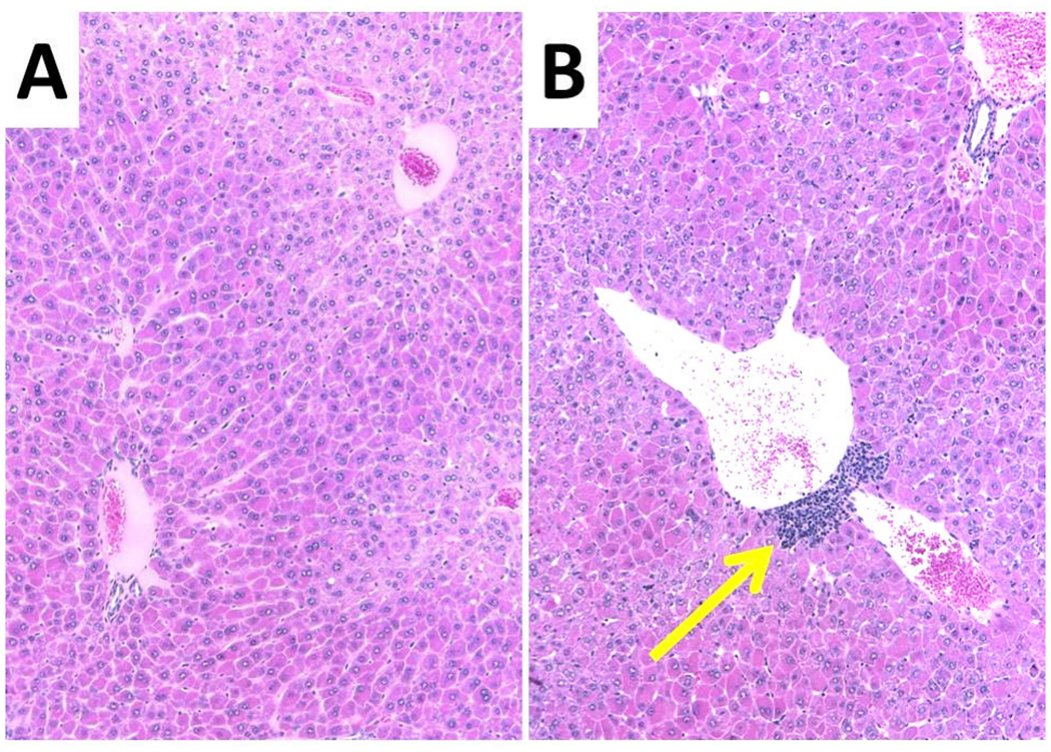
Hematoxylin and eosin (H&E) staining of representative liver samples from WY-14,643 treated C3H/HeJ (panel A) and C57BL/6 (panel B). Sinusoidal cellular infiltrate are high-lighted with a yellow arrow.

Finally, the differential immune response of the two strains was confirmed through H&E staining of liver samples. Fig 6 shows representative images of treated livers from each strain. Increased sinusoidal infiltrates were clearly visible in all C57BL/6 samples (Fig 6 and Table 3).

**Table 3:** Graded histopathologic changes in individual animals.

Strain	C3H/HeJ Strain								C57BL/6							
Treatment	Control				WY-14,643				Control				WY-14,643			
Mouse ID#	31	32	33	34	51	52	53	55	37	38	39	40	56	57	59	60
Hypertrophy, hepatocyte	0	0	0	0	4	4	4	4	0	0	0	0	4	4	4	4
Mitotic figures, Hepatocyte	0	0	0	0	2	2	2	2	0	0	0	0	2	2	2	2
Cellular infiltrate, sinusoidal	0	0	0	0	0	0	0	0	0	0	0	0	1	2	1	1

## Discussion

A number of groups have reported that retroelements are 100X more active within mice than they are in humans. In addition, analyses of spontaneous mutations within both species indicates that retrotranspositional events accounts for 10% of all mutations in mice but less than 0.1% in humans [12, 41]. One of the major factors controlling transcription of these retroelements is DNA methylation with a demethylated state leading to an increase in transcription of many of these elements [20].

Many retroelements are still competent for retrotransposition in mice, which presents the possibility that reactivation of these elements and the resulting random chromosomal insertional mutations are what drives the non-genotoxic carcinogenesis observed with WY-14,643. In addition to potential new retrotransposition events, other workers have shown that activation of retroelements can affect expression of nearby genes by providing promoters or enhancers or producing long non-coding RNAs [11, 42]. Indeed, it is interesting to note that VL30 elements, a family of mouse ERVs, appears to be among the most highly up-regulated families in the WY-14,643 treated mice. De-repression of these elements in mouse hepatocytes has been previously shown to cause deregulation of neighboring genes and is also linked to activation of viral-defense responses which resemble a pre-neoplastic inflammatory state [42].

In this report, we have shown that the PPARα agonist WY-14,643 alters the expression of a number of retroelements within C3H/HeJ and C57BL/6 mice. We have also shown that a significant number of LTR and LINE1 elements exhibit increased expression with WY-14,643 treatment. This result is consistent with a report from Pogribny et al who reported that WY-14,643 induces the DNA demethylation of these element classes [10].

Interestingly, both the DNA demethylation and the carcinogenicity activity are dependent on PPARα activity implying a linkage between the two [10, 43]. Another point implicating retrotransposition in the observed tumorigenicity is that while PPARα activators are tumorigenic in rodents, they have been deemed of limited to no risk for liver cancer in humans [5, 44, 45].

While the retroelement transcriptional data described in this report supports retrotransposition or other retroelement-mediated effects as potential mechanisms for the observed non-genotoxic carcinogenicity, they do not explain the differences in susceptibility seen between the mouse strains. It is also worth noting that more germ-line mutations due to LTR retroelement insertions have occurred in C3H-related strains, compared to other common strains [12]. To determine if there are any fundamental differences between these two strains, we compared the differential expression of the untreated liver samples from C57BL/6 to the untreated samples from C3H/HeJ. From this analysis, we found a number of immune activities to be significantly higher in the tumor resistant strain. In addition, Yu *et al*, demonstrated Toll-like receptors are essential for the control of endogenous retroviruses in C57BL/6 [46]. These results led us to examine the known genetic background of these two strains. Of particular note is that the C3H/HeJ strain carries a mutation in the toll-like receptor 4 (Tlr4^Lps-d^). Mice with this mutation are highly susceptible to infection particularly with gram-negative bacteria [47]. These mutant mice also show reduced chemokine production, and an overall attenuated cellular immune response [48]. In addition to detecting bacterial proteins, the TLR4 receptor has been demonstrated to respond to retroviral envelop (env) proteins [49, 50]. Since many retroelements encode for an env protein which could reach the cell surface and hence be detected by TLR4, it is feasible that Wyeth-14,643 induced retroelements could elicit a TLR4 mediated response. To explore this possibility we compared the effects of Wyeth-14,643 treatment on gene expression between the two strains. For both strains, the predominant effect was a strong drug response in many of the pathways related to the therapeutic activity of this PPARα-agonist. However, only the less tumor sensitive C57BL/6 exhibited a robust antiviral response with WY-14,643 treatment.

To confirm this strain specific immune response, we histologically examined liver samples from both strains pre- and post-treatment with WY-14,643. Treated livers from both C3H/HeJ and C57BL/6 livers displayed increased hepatocyte hypertrophy and mitosis consistent with the reported liver effects of PPARα activators [5, 44]. However, only the five samples from the WY-14,643-treated C57BL/6 liver exhibited increased sinusoidal cellular infiltrates (Fig 6 and Table 3).

These results suggest that only the C57BL/6 strain is exhibiting a robust immune response to the transcriptional activation of its retroelements. An attenuated immune response in the C3H/HeJ strain would leave it susceptible to the mutagenic activity of these retroelements when they randomly recombine into the chromosome. In agreement with this hypothesis, we observed that Ingenuity reports increased “recombination” functions for the liver samples from the WY-14,643 treated C3H/HeJ strain but not from any others (Fig 4B). A stronger immune response in C57BL/6 to basal retroviral activity may also explain the trend towards higher hepatocarcinogenesis rates in the C3H/HeJ strain versus the C57BL/6 strain observed in the absence of added carcinogen [51].

## Conclusions

In this report, we investigated the role that retroelement reactivation may play in compound-driven mouse specific carcinogenesis. We used the well-studied mouse nongenotoxic carcinogen WY-14,643. We show that this compound induced a general increase in LTR and LINE retroelements, two retroelement classes known to be highly active in mice and to induce the majority of all retroelement driven mutations in mice [12, 41]. Our results are also in-line with work done by Pogribny, who showed that WY-14,643 causes the demethylation of these elements [10]. Given that others have shown that retroelement mutational activity is at least 100X more active in mice than it is in humans [12], our results could explain the mouse-specific carcinogenic nature of WY-14,643. In mice, retroviral reactivation would lead to retranspositional events, genomic mutation and ultimately to carcinogenesis. Interestingly retroelement reactivation may play a role in some auto-immune diseases in humans. It has been shown that endogenous retrovirus reactivation may play a role in multiple sclerosis and other neuroinflammatory diseases [52, 53].

The retroelement expression data alone does not explain why there should be such significant differences in the rate of WY-14,643 driven carcinogenesis between the two strains. While we cannot rule out the possibility that individual retroelements having higher mutagenic capacity being more elevated in the tumor-prone C3H/HeJ, the basal gene expression differences between C3H/HeJ and C57BL/6 reveals a clear difference in immune system activity. This difference likely arises from the fact that the C3H/HeJ strain has an inactivating mutation in its Tlr4 gene. Since Tlr4 is known to have the ability to sense retroviral env protein, the C57BL/6 strain likely is capable of responding to and suppressing the activity of cells expressing retroelement components, including env. We see clear evidence of this retroelement response in the resistant C57BL/6 strain when the animals are treated with WY-14,643. Expression analysis indicates that a number of genes involved in retroviral infection are elevated in this strain with treatment. The same response is not seen in the tumor-prone C3H/HeJ strain. We also saw a clear strain-specific immunologic difference by histology with WY-14,643 treatment.

Among the antiviral signals we observed in the resistant C57BL/6 was a clear change in the expression of a number of genes in the “mitochondrial dysfunction” pathway. We interpreted this change as part of the innate immune response. It is becoming increasingly clear that one of the mitochondria’s many roles is in the innate immune response [38]. The immune function role for the mitochondria could also explain a previous report by Isenberg et al., who showed that rotenone inhibited the formation of hepatic lesions with WY-14,643 in mice [54]. Rotenone has long been known to exhibit antiviral activity possibly through an enhancement of innate immune cell killing of virally infected cells by increasing mitochondrial reactive oxygen species [55, 56]. A similar enhancement of the antiviral activity by rotenone would explain the reduced number of hepatic lesions when WY-14,643 treated mice were co-treated with rotenone [54].

In conclusion, WY-14,643 clearly increases the expression of retroelements known to be retrotranspositionally active in mice. This increased expression could explain the mouse-specific nongenotoxic tumorigenesis via either insertional mutagenesis or other gene regulatory effects of transcriptionally activated retroelements. Given that a number of nongenotoxic mouse-specific carcinogens alter DNA methylation [9], this mechanism may play a more general role in mouse-specific compound-induced carcinogenesis. And may provide a path forward into human for promising new drugs which otherwise might be terminated due to a tumor finding in mice. Inter-strain variation in antiviral immune-response would, in turn, lead to differential rates of tumorigenesis may suggest a further avenue to explore when a new therapeutic is suspected to be a mouse-specific carcinogen.

## Supporting information

S1 Excel File Expression values for LTR and LINE elements. Individual FPKM expression values for each animal, average expression fold-change and t-test values for each condition and charts of—log10 of p-values versus fold-change.
(XLSX)

S2 Excel File Analyses of differentially expressed genes between untreated strains. IPA pathway enrichment and upstream analysis of gene expression. Analyses were performed on differentially expressed genes in the untreated C3H/HeJ versus the untreated C57BL/6 strains.
(XLS)

S3 Excel File Analyses of differentially expressed genes between WY-14643-treated C57BL/6 and untreated C57BL/6. IPA’s canonical pathway, upstream regulator, disease & biofunctions, and tox functions analyses were performed on genes identified as differentially expressed between the WY-14643 treatment and the control animals in the C57BL/6 strain.
(XLS)

S4 Excel File Analyses of differentially expressed genes between WY-14643-treated C3H/HeJ and untreated C3H/HeJ. IPA’s canonical pathway, upstream regulator, disease & biofunctions, and tox functions analyses were performed on genes identified as differentially expressed between the WY-14643 treatment and the control animals in the C3H/HeJ strain.
(XLS)

S5 Excel File Gene expression values for all samples and time points. Individual RPKM expression values for each animal, average expression fold-change and t-test values for each condition.
(XLSX)
